# A Distinct miRNA Profile in Intimal Hyperplasia of Failed Arteriovenous Fistulas Reveals Key Pathogenic Pathways

**DOI:** 10.3390/biom15081064

**Published:** 2025-07-23

**Authors:** Carmen Ciavarella, Francesco Vasuri, Alessio Degiovanni, Lena Christ, Raffaella Mauro, Mauro Gargiulo, Gianandrea Pasquinelli

**Affiliations:** 1 Department of Medical and Surgical Sciences (DIMEC), University of Bologna, 40138 Bologna, Italy; francesco.vasuri2@unibo.it (F.V.); lena-christ@gepromed.com (L.C.); mauro.gargiulo2@unibo.it (M.G.); gianandr.pasquinelli@unibo.it (G.P.); 2 Pathology Unit, Santa Maria Delle Croci Hospital, 48121 Ravenna, Italy; 3 Pathology Unit, IRCCS Azienda Ospedaliero-Universitaria di Bologna, 40138 Bologna, Italy; alessio.degiovanni@aosp.bo.it; 4 Vascular Surgery Unit, IRCCS Azienda Ospedaliero-Universitaria di Bologna, 40138 Bologna, Italy; raffaella.mauro1@gmail.com

**Keywords:** miRNA, intimal hyperplasia, arteriovenous fistula failure, proliferation, migration, hsa-miR-449a-5p

## Abstract

Intimal hyperplasia (IH) compromises the patency of arteriovenous fistula (AVF) vascular access in patients with end-stage kidney disease. Uncontrolled cell proliferation and migration, driven by inflammation, shear stress and surgery, are well-known triggers in IH. Recently, microRNAs (miRNAs) have emerged as regulators of core mechanisms in cardiovascular diseases and as potential markers of IH. This study was aimed at identifying a specific miRNA panel in failed AVFs and clarifying the miRNA involvement in IH. miRNA profiling performed in tissues from patients with IH (AVFs) and normal veins (NVs) highlighted a subset of four miRNAs significantly deregulated (hsa-miR-155-5p, hsa-miR-449a-5p, hsa-miR-29c-3p, hsa-miR-194-5p) between the two groups. These miRNAs were analyzed in tissue-derived cells (NVCs and AVFCs), human aortic smooth muscle cells (HAOSMCs) and human umbilical vein endothelial cells (HUVECs). The panel of hsa-miR-449a-5p, hsa-miR-155-5p, hsa-miR-29c-3p and hsa-miR-194-5p was up-regulated in AVFCs, HAOSMCs and HUVEC under inflammatory stimuli. Notably, overexpression of hsa-miR-449a-5p exacerbated the proliferative, migratory and inflammatory features of AVFCs. In vitro pharmacological modulation of these miRNAs with pioglitazone, particularly the down-regulation of hsa-miR-155-5p and hsa-miR-29c-3p, suggested their involvement in IH pathogenesis and a potential translational application. Overall, these findings provide new insights into the pathogenesis of AVF failure, reinforcing the miRNA contribution to IH detection and prevention.

## 1. Introduction

Intimal hyperplasia (IH) is an occlusive lesion that leads to the progressive narrowing of the vascular lumen and typically occurs following vascular surgery [1,2]. IH affects the patency of arteriovenous fistula (AVF), which is the gold-standard vascular access for hemodialysis in chronic kidney disease (CKD) patients, leading to increased morbidity and mortality rate [3]. The surgical trauma following the AVF procedure, the shear stress and the patient clinical setting (i.e., uremia, hypertension) are predisposing factors to IH. Indeed, these conditions stimulate inflammation, hypoxia and oxidative stress that interfere with the physiological remodeling and the correct maturation of AVF, leading to outward remodeling and wall degeneration. Current research is addressed at elucidating the molecular complexity governing IH pathogenesis and AVF failure. The transcriptomic profile of human AVF determined by RNA sequencing highlighted the up-regulation of pro-inflammatory genes co-localizing with vascular smooth muscle cells (VSMCs) in failed AVF [4]. VSMCs dedifferentiate from a contractile to a synthetic phenotype under inflammatory stimuli, undergoing unbalanced proliferation, migration to the intima and matrix remodeling, typical hallmarks of IH [5]. In this regard, a significant association between intimal thickening and Ki-67 expression, a robust cell proliferation marker, was shown in AVF tissues [6]. A noteworthy contribution to IH derives from endothelial cells (ECs) that lose endothelial nitric oxide synthetase (eNOS) following vascular damage, leading to NO reduction and VSMC dedifferentiation. The importance of eNOS as a potential therapeutic target has been recently underlined in a rat model of AVF [7]. Furthermore, ECs are involved in vascular remodeling by phenotype switching into mesenchymal-like cells through the well-known endothelial-to-mesenchymal transition (End-MT) process [8]. Several pathways trigger End-MT, especially under disturbed shear stress, including Extracellular Signal Regulated Kinase (ERK)-5 signaling [8] and the osteopontin/CD44 axis [9]. End-MT inhibition in a mouse vein graft model was shown to reduce IH and AVF stenosis [10].

Increasingly evidence supports the orchestrating role of micro-RNAs (miRNAs) in vascular pathophysiology. miRNAs are a class of small non-coding RNAs (18–22 nucleotides in length) that finetune gene expression at a post-transcriptional level, hence regulating many steps of cell proliferation, migration, differentiation and inflammation, key mechanisms of IH disease [11]. miR-155 promotes neointima formation in a mouse model of AVF by up-regulating the inflammatory cascade [12]. The serum exosome miR-200a-3p increases IH via VEGF and Ang-II [13]. Up-regulation of miR-21 was seen in stenotic veins of patients’ AVFs, correlating with a high risk of restenosis [14]. Interestingly, a recent study evidenced an altered miRNA profile in stenotic AVFs of CKD patients, identifying six miRNAs significantly different between normal and stenotic veins [15]. The authors also highlighted through a bioinformatic approach the potential pathogenic role of the mitogen-activated protein kinases in an AVF failure process [15].

The present study was aimed at investigating the miRNA involvement in IH and AVF failure. To reach this goal, tissue and cell cultures obtained from AVFs of CKD patients were collected. A miRNA profile was assessed in AVF tissues compared to NV tissues, leading to the identification of a miRNA subset specific to AVF with IH lesions. Then, to deepen the biological relevance of the most significant miRNAs that emerged from the first phase of the study, we analyzed their expression pattern in human vascular cell models, focusing on hsa-miR-449a-5p and its potential contribution to IH pathogenesis by ectopic over-expression.

## 2. Materials and Methods

### 2.1. Study Design and Study Population

The present study was performed on patient-derived tissue and cell models. Vascular tissues were collected from patients with end-stage kidney failure subjected to an AVF surgical procedure, under the approval of the Ethical Committee of the Emilia-Romagna Region (protocol number 142/2019/Sper/AOUBo). All tissue samples were treated anonymously and according to the ethical guidelines of the 1975 Declaration of Helsinki and following revisions. Tissue samples were distinguished as follows: (1) normal veins (NVs) collected from pre-dialytic patients subjected to the first AVF surgery, and (2) AVFs, arteriovenous fistulae tissues with IH collected from patients undergoing AVF revision. Each sample was in part stored at −80 °C for RNA extraction and in part processed for organ culture and cell isolation, as previously described [16].

### 2.2. microRNA Expression Profiling in Human AVF Tissues

miRNA profiling was performed in 4 NV and 4 AVF to identify a miRNA subset specific for failed AVF. Samples previously stored at −80 °C were processed for total RNA extraction by homogenization in TRIzol Reagent (Thermo Fisher Scientific, USA), according to the manufacturer’s instruction. The RNA quantity and purity were analyzed by a ND-1000 spectrophotometer (NanoDrop, Thermo Fisher Scientific, USA). RNA purity was assessed by measuring the absorbance ratio with proteins (260/280, range: 1.8–2.1) and phenol contaminants (230/280, range: 2–2.2). Reverse transcription was performed using the TaqMan^®^ MicroRNA Reverse Transcription Kit and Megaplex RT Primers Pool A (Thermo Fisher Scientific, USA). A pre-amplification step was performed by combining 2.5 μL of the RT reaction with the matching Megaplex PreAmp Primer Pool and TaqMan PreAmp Master Mix (Thermo Fisher Scientific, USA) under the following conditions: 10 min at 95 °C; 2 min at 55 °C; 2 min at 72 °C; 15 s at 95 °C and 4 min at 60 °C for 12 cycles; and 99 °C for 10 min. Reverse transcription and pre-amplification reactions were carried in the Biorad T100 Thermal Cycler (BioRad Laboratories Hercules, CA, USA S.r.l.). The miRNA profiling was carried out with 384-well TaqMan Human microRNA arrays (Gene expression Micro Fluidic card, Array A v2.1, Life Technologies Waltham, MA, USA) and run on a 7900 HT Real Time System (Life Technologies) with the following conditions: 10 min at 95 °C, 15 s at 95 °C and 1 min at 60 °C for 40 cycles. Raw data were analyzed with Expression Suite software v1.0 (Thermo Fisher Scientific), and the comparative Ct method (∆∆CT) was used [17]. Ct values < 40 were established as a cut-off, and fold changes (FC, 2^−∆∆CT^) ≥ 2 and ≤−2 were selected. The non-coding small nuclear RNA gene U6 (snU6) was used as a reference gene.

### 2.3. Validation of miRNA Expression by Real-Time PCR

Following the profiling step, we selected a panel of 4 miRNAs significantly deregulated between NV and AVFs for validation. To this end, total RNA was extracted from 16 tissues (8 NV and 8 AVFs) by homogenization in TRIzol Reagent (Thermo Fisher Scientific), according to the manufacturer’s instruction. A pool of primers/probes specific for the target miRNAs (hsa-miR-155-5p, hsa-miR-449a-5p, hsa-miR-29c-3p, hsa-miR-194-5p) was prepared for reverse transcription (TaqMan^®^ MicroRNA Reverse Transcription Kit, Thermo Fisher Scientific, USA) and pre-amplification (TaqMan^®^ PreAmp Master Mix, Thermo Fisher Scientific, USA). miRNA expression was evaluated by Real-Time PCR with the TaqMan MicroRNA Assay using specific probes for each target and TaqMan Universal Master Mix II No AmpErase UNG (Thermo Fisher Scientific, USA). The miRNA assay ID and sequences are reported in Supplementary Table S1. The reaction was carried out in BioRad CFX Connect (Biorad Laboratories S.r.l.). Each assay was executed in triplicate, and small nuclear gene U6 was used as a reference gene (Assay ID: 001973; Thermo Fisher Scientific, USA). The relative expression was determined by the comparative 2^−ΔΔCt^ method [17], and data were expressed as fold changes relative to NV. To explore the biological role of selected miRNAs, we explored the putative pathways involving the selected miRNAs by mirPath v.4 (https://dianalab.e-ce.uth.gr/html/mirpathv3/index.php?r=mirpath, accessed on 16 June 2025) [18] and miRNET [19].

### 2.4. Cell Models of AVF Pathogenesis and Treatments

In the present study, we used three different cell models to study the miRNA involvement in AVF pathogenesis: primary vascular cell cultures, aortic smooth muscle cells and endothelial cells. Normal vein cells (NVCs) were isolated from patients’ native veins, and AVF cells (AVFCs IH) were isolated from AVF patients. Briefly, tissues were rinsed with phosphate-buffered saline (PBS, Merck Group, Darmstadt, Germany) and cultured with growth medium (Dulbecco’s Modified Eagle Medium with Glutamax (DMEM, Thermo Fisher Scientific) enriched with 20% FBS and 1% antibiotics) in 12-well plates. Tissue cultures were kept in an incubator at 37 °C and 5% CO_2_ for two weeks, and growth medium was freshly replaced every three days. After tissue removal, cells were cultured for up to 10 days, when 70% confluence was reached. The cell immunophenotype and proliferation have been previously characterized [16]. Human aortic smooth muscle cells (HAOSMCs) and human umbilical vein endothelial cells (HUVECs) were purchased from Promocell (Heidelberg, Germany) and cultured in smooth muscle growth medium 2 and endothelial growth medium, respectively (Promocell, Heidelberg, Germany). HAOSMCs and HUVECs were treated with the inflammatory cytokine tumor necrosis factor-α (TNF-α; PeproTech, Cranbury, NJ, USA) at 25 ng/mL for 1 week to mimic the pathological setting of AVF and IH lesions. Pioglitazone hydrochloride (Merck Life Science, Darmstadt, Germany) was administrated at 10 μM for 48 h in AVFCs in growth medium and in HAOSMCs and HUVECs cultured with TNF-α.

### 2.5. Cell Transfection

For hsa-miR-449a-5p over-expression in AVFCs, the mirVana miRNA mimic (Thermo Fisher Scientific, Waltham, MA, USA) and Lipofectamine RNAiMAX (Thermo Fisher Scientific, Waltham, MA, USA) were used according to the manufacturer’s instructions. The mimics used were hsa-miR-449a (hsa-miR-449a, assay ID: MC11127, mature miRNA sequence: UGGCAGUGUAUUGUUAUCUGGU) and a mimic negative control (miR-NC) (10 pmol/µL). After transfection, cells were processed for RNA extraction, proliferation and migration assays.

### 2.6. miRNA Expression Analysis in Vascular Cell Models

The miRNA subset emerged from tissue study was analyzed in vascular cell models. To this end, total RNA was extracted from cell cultures using TRIzol Reagent (Thermo Fisher Scientific, Waltham, MA, USA), according to the manufacturer’s instructions. cDNA reverse transcription was performed with miRNA-specific primers in the same reaction using TaqMan microRNA Assays and a TaqMan microRNA Reverse Transcription Kit (Thermo Fisher Scientific, Waltham, MA, USA), following the manufacturer’s instructions. miRNA expression was performed through Real-Time PCR, using TaqMan MicroRNA Assay-specific probes for each target (Supplementary Table S1) and TaqMan Universal Master Mix II No AmpErase UNG as described above. Results were determined by the comparative 2^−∆∆Ct^ method and expressed as fold changes relative to controls.

### 2.7. Analysis of Cell Proliferation

Cell proliferation was evaluated by immunofluorescence staining of Ki-67 and a bromo-deoxy-uridine (BrdU) assay. For Ki-67 staining, cells were fixed with 4% paraformaldehyde for 4 min at rt and permeabilized with Triton X-100 (Merck Group, Darmstadt, Germany) at 1% in PBS for 10 min at rt. Incubation with 1% bovine serum albumin (BSA, Merck Group, Darmstadt, Germany) in PBS was performed for 30 min at rt for the blocking of non-specific binding sites. Incubation with anti-ki-67 primary antibody (1:100, Novocastra, Leica Biosystems, Wetzlar, Germany) was performed for 1 h at 37 °C, followed by PBS washes and incubation with anti-mouse Alexa Fluor 488 secondary antibody (ThermoFisher Scientific, Carlsbad, CA, USA) in 1% BSA/PBS for 1 h at 37 °C in the dark. After PBS washes, cells were stained with DAPI (4′, 6-diamidino-2-phenylindole; Thermo Fisher Scientific, Carlsbad, CA, USA) for nuclei detection. Images were acquired and analyzed by a Leica DMI4000 B inverted fluorescence microscope (Leica Microsystems, Milan, Italy). Quantification of Ki-67-positive cells was performed on digitalized images randomly acquired at a 20× magnification: positivity was expressed as the percentage of positive nuclei/total cells. For BrdU, cells were seeded into a 96-well plate at a density of 2 × 10^4^/well. After 24 h, miRNA and siRNA transfections were performed as described elsewhere in this study. After 48 h, a BrdU cell proliferation assay was performed according to the manufacturer’s protocol (Millipore), and cellular BrdU incorporation was measured by absorbance analysis at a 450 nm optical density (OD) with a reference wavelength of 595 nm by a Spark multimode microplate reader (Tecan, Zurich, Switzerland).

### 2.8. Scratch Wound Assay

The effect of miR-449a-5p mimic was evaluated in terms of cell migration in AVFCs. Cells were seeded in an Incucyte ImageLock 96-well plate (Sartorius) and exposed to transfection mix for 4 h. Before changing the medium, the automated cell scratch was performed through the WoundMaker (Essen BioScience, Ann Harbor, MI, USA) device. The plate was placed in the IncuCyte S3 instrument (Essen BioScience, Ann Harbor, MI, USA) equipped with a dedicated incubator. Each wound image per well was automatically recorded with a 10× objective lens every 3 h for 48 h using the IncuCyte S3/SX1 optical module phase contrast. Image analysis was performed by measuring the following metrics: the wound width, which is the distance between the migrating edges of the wound expressed in micrometers (μm), and the wound confluence, which is the wound area covered by cells expressed as a percentage (%).

### 2.9. Inflammatory Cytokine Detection

To verify the contribution of hsa-miR-449a-5p to the inflammatory setting in AVF, we analyzed levels of interleukins (IL-1β, IL-6, IL-8) and tumor necrosis factor α (TNF-α) in surnatants of AVFCs IH. Briefly, cell culture mediums were collected from AVFCs after 48 h from hsa-miR-449a-5p over-expression, centrifuged for debris removal and stored at −80 °C until use. For cytokine detection, the fully automated immunoassay platform ELLA (Protein Simple/Bio-techne, San Jose, CA, USA) was used, according to the manufacturer’s instructions. Final data are reported as the mean ± standard deviation of three technical replicates obtained from two independent experiments and expressed as pg/mL.

### 2.10. Statistical Analysis

Data analysis and statistical analysis relative to miRNA profiling data were performed by Expression Suite software v1.0 (Thermo Fisher Scientific). RT-qPCR, cell transfection, and relative assays were executed at least in three biological and technical replicates, and data are expressed as mean ± standard deviation (SD). Statistical analysis was performed using GraphPad Prism 8, and the following tests were used: unpaired Student’s t test for comparison between two groups after normality test evaluation and two-way analysis of variance (ANOVA) for comparison with more than two groups followed by Sidak’s post hoc test for multiple comparisons. The null hypothesis was rejected with a *p* value <0.05 (in a confidence interval of 95%).

## 3. Results

### 3.1. Study Population and Clinical Data

The present study recruited vascular tissues from 16 end-stage kidney disease patients subjected to AVF surgery. The study population was divided in two main groups: NV (patients enrolled at the first AVF surgery, without histological alterations of normal vein tissue) and AVF (patients underwent revision of previous AVF). The AVF group included both veins carrying IH lesions and those with aneurysmal wall degeneration. All the patients underwent multiple drug therapy for the chronic disease, without significant differences in the therapeutic protocol between the two groups. The demographic and clinical data of the study population are detailed in Table 1.

### 3.2. miRNA Profiling Highlights a Subset of miRNAs Differently Expressed Between Normal and IH Veins from CKD Patients

**Figure 1:**
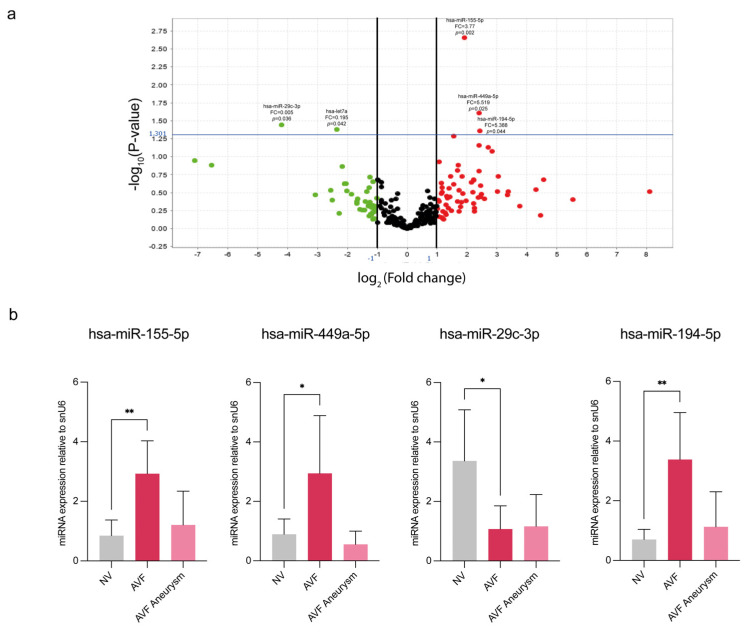
Expression profile of miRNAs in AVFs compared to normal veins. (**a**) Volcano plot representation of the miRNA profile in human AVFs compared with NV. Data are reported as fold changes (log_2_) versus *p* value (−log_10_). Green points: down-regulated miRNAs; red points: up-regulated miRNAs. Fold change boundary: 2; *p*-value: 0.05 (points above the horizontal line are miRNAs significantly deregulated). The graphic representation was obtained with Expression Suite Software v1. (**b**) Validation by RT-qPCR of selected miRNAs (hsa-miR-155-5p, hsa-miR-449a-5p, hsa-miR-29c-3p, hsa-miR-194-5p) in AVF veins compared to the NV group. The AVF group was further divided in two subgroups: AVF IH, carrying intimal hyperplasia; AVF aneurysm, with aneurysmal degeneration of the vascular wall. Small nuclear RNA U6 (snU6) was selected as an endogenous control, and the relative miRNA expression was calculated with the 2^−∆∆CT^ method. Data are expressed as mean ± SD, and statistical analysis was performed by unpaired Student *t* test; * *p* < 0.05, ** *p* < 0.01. NV, normal veins; AVF, arteriovenous fistula; FC, fold changes.

To investigate the involvement of miRNAs in the pathogenesis of intimal hyperplasia and consequent failure of AVF, preliminary profiling was performed on eight sample tissues (four NVs and four AVFs) by microfluidic cards. The analysis was performed by comparing AVF tissues versus NVs and revealed a total of 288 miRNAs detected across all samples, as illustrated in the volcano plot (Figure 1a). Out of 288, 101 miRNAs were down-regulated and 136 were up-regulated in AVF (Supplementary Table S2). A group of five miRNAs was significantly deregulated in AVFs versus NVs: hsa-miR-155-5p, hsa-miR449a, hsa- hsa-miR-194b-5p (up-regulated), hsa-miR-29c-3p and let7a (down-regulated).

Based on statistical analysis, we selected hsa-miR-155-5p, hsa-miR449a-5p, hsa-miR-29c-3p and hsa-miR-194-5p for validation by RT-qPCR in 16 samples (eight NVs, eight AVFs). The AVF group was further divided in two subgroups: AVF IH and AVF aneurysm, to distinguish between early IH lesions and late vascular degeneration following aneurysm dilation. The validation step confirmed the profiling data, elucidating the most significant differences in miRNA expression between the NV and AVF IH groups (Figure 1b). The results obtained from miRNA profiling and RT-qPCR validation are summarized in Table 2.

To gain insight into the biological role of the selected miRNAs, we performed an analysis of the KEGG (Kyoto Encyclopedia of Genes and Genomes) pathways with the bioinformatic database mirPATH v.4.0 using the DIANA tool, by applying the Tarbase algorithm with FDR correction and a *p*-value threshold of 0.05. According to the pathway union analysis (Supplementary Table S3), among the most significant pathways that differently involved at least 1–2 of the selected miRNAs, focal adhesion (*p* 4.11 × 10^−12^), the p53 signaling pathway (*p* 1.18 × 10^−9^), the FoxO signaling pathway (*p* 6.67 × 10^−9^), the adherens junction (*p* 2.87 × 10^−6^) and the Hippo signaling pathway (*p* 9.92 × 10^−6^) emerged. In addition, miR-29c-3p was also involved in the fluid shear stress and atherosclerosis pathway (*p* 3.35 × 10^−5^) and the TNF signaling pathway (*p* 0.003). The enrichment analysis in the GO:BP dataset highlighted the negative regulation of transcription by the RNA polymerase II (*p* 9.088 × 10^−24^) and the chromatin organization (*p* 8.62248 × 10^−23^) pathways involving all the candidate miRNAs. The complete analysis performed by miRPATH v4.0 using the DIANA tool and miRNET are reported in Supplementary Tables S3–S6.

### 3.3. Analysis of the AVF IH miRNA Subset in Human Vascular Cell Models

**Figure 2:**
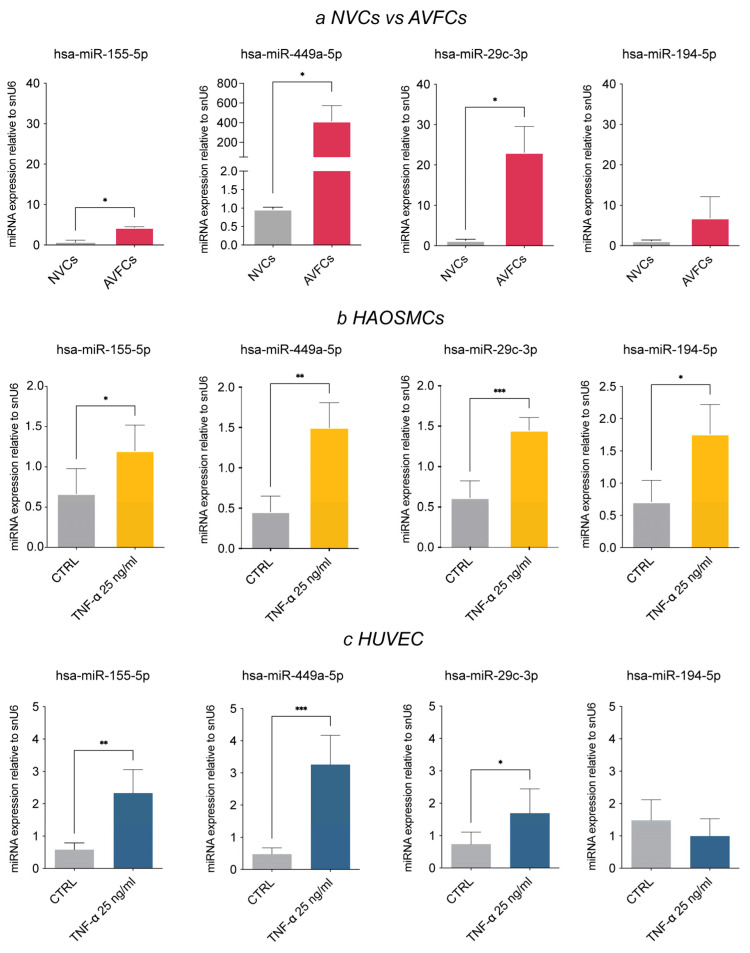
The miRNA expression pattern in human vascular cells mirrors the AVF tissue signature. RT-qPCR for hsa-miR-155-5p, hsa-miR-449a-5p, hsa-miR-29c-3p and hsa-miR-194b was performed in (**a**) AVFCs compared to NVCs, (**b**) HAOSMC and (**c**) HUVEC exposed to chronic inflammatory injury by TNF-α administration for 6 days (25 ng/mL) compared to untreated controls. Small nuclear RNA U6 (snU6) was selected as endogenous control, and the relative miRNA expression was calculated with the 2^−∆∆CT^ method. Data are reported as mean ± SD of at least three independent experiments; * *p* < 0.05, ** *p* < 0.01, *** *p* < 0.001; unpaired Student *t*-test.

The second part of this study was performed on human vascular cell models to unveil the role of the selected miRNAs in IH pathogenesis. To this aim, we firstly explored the expression status of the miRNA panel in patient-derived cells (NVCs vs. AVFCs) and smooth muscle/endothelial cell models (HAOSMCs and HUVECs), which were primed with TNF-α for 6 days to mimic the pathological setting of a long inflammatory injury. The miRNA expression pattern in cell models agreed with patient tissues, except for hsa-miR-29c-3p. Indeed, hsa-miR-155-5p, hsa-miR449a, hsa- miR-29c-3p and hsa-miR-194-5p were all up-regulated in both AVFCs IH (compared to NVCs; Figure 2a) and in TNF-α-primed HAOSMCs (compared to untreated controls; Figure 2b).

### 3.4. Pioglitazone, a PPAR-γ Agonist, Modulates miRNA Expression Levels In Vitro

**Figure 3:**
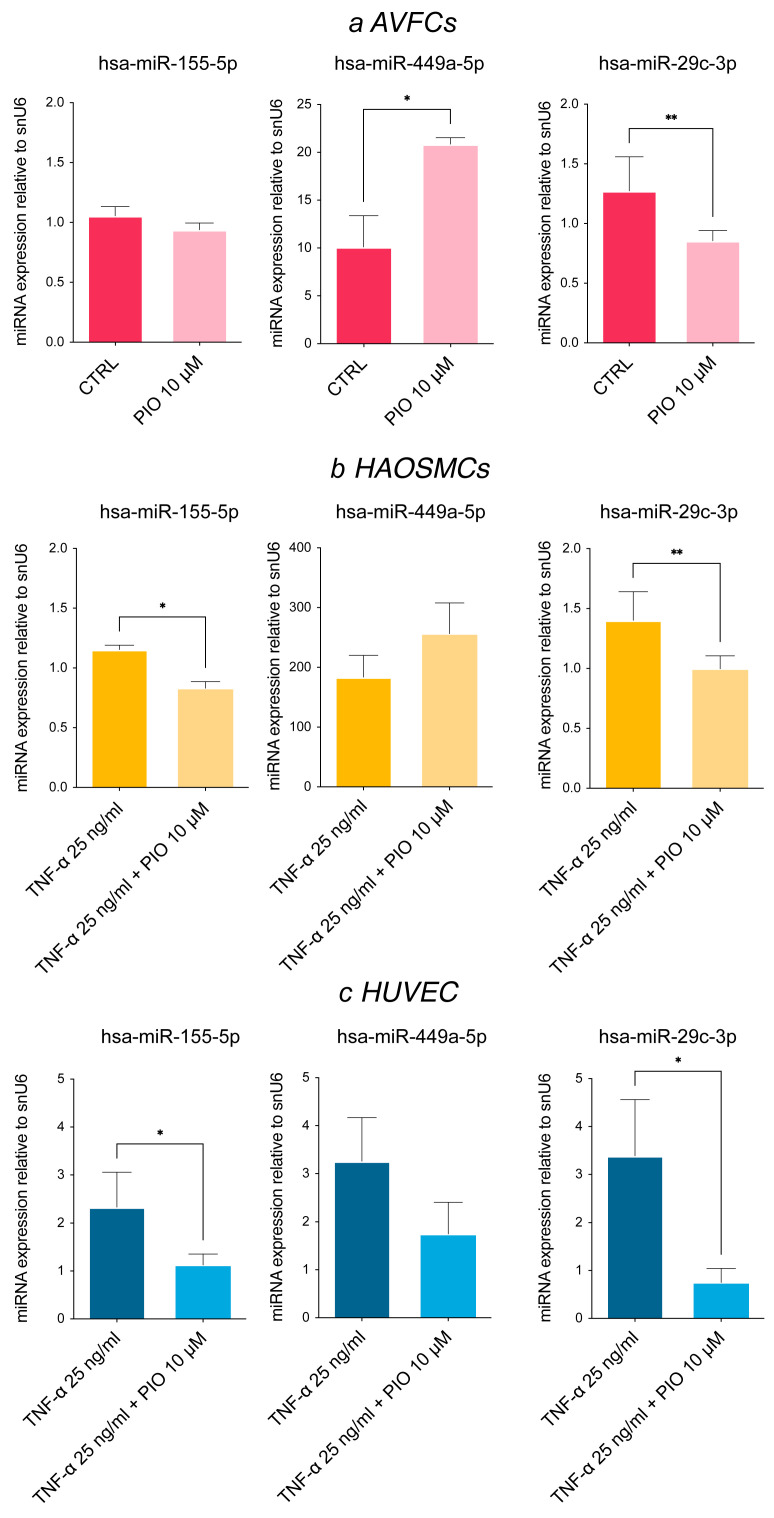
Pioglitazone modulates IH-associated miRNA expression in vascular cell models. Pioglitazone differently modulates the IH-miRNA expression levels in (**a**) AVFCs, (**b**) HAOSMCs and (**c**) HUVEC, by down-regulating the levels of hsa-miR-29c-3p and hsa-miR-155-5p and up-regulating hsa-miR-449a-5p in AVFCs. Results are expressed as fold changes relative to untreated controls. Small nuclear RNA U6 (snU6) was selected as endogenous control, and the relative miRNA expression was calculated with the 2^−∆∆CT^ method. Data are reported as mean ± SD of at least three independent experiments; * *p* < 0.05, ** *p* < 0.01, unpaired Student *t*-test.

The regulatory role of pioglitazone on cell proliferation and migration via PPAR-γ activation led us to explore possible pioglitazone modulation of the identified miRNA panel. To this end, AVFCs, TNF-α-primed HAOSMCs and TNF-α-primed HUVECs were exposed to pioglitazone, demonstrating a significant down-regulation of hsa-miR-155-5p and hsa-miR-29c-3p (Figure 3a–c). These data deepen our knowledge of the pioglitazone mechanism, adding novel insights into its beneficial properties by normalizing the expression levels of miRNAs involved in IH pathogenesis. Conversely, we found a significant up-regulation of hsa-miR-449a-5p in both AVFCs T1 and TNF-primed HAOSMCs following drug treatment (Figure 3a,b), pointing to an alternative regulatory mechanism.

### 3.5. miR-449a-5p Promotes Cell Proliferation, Migration and the Inflammatory Process

**Figure 4:**
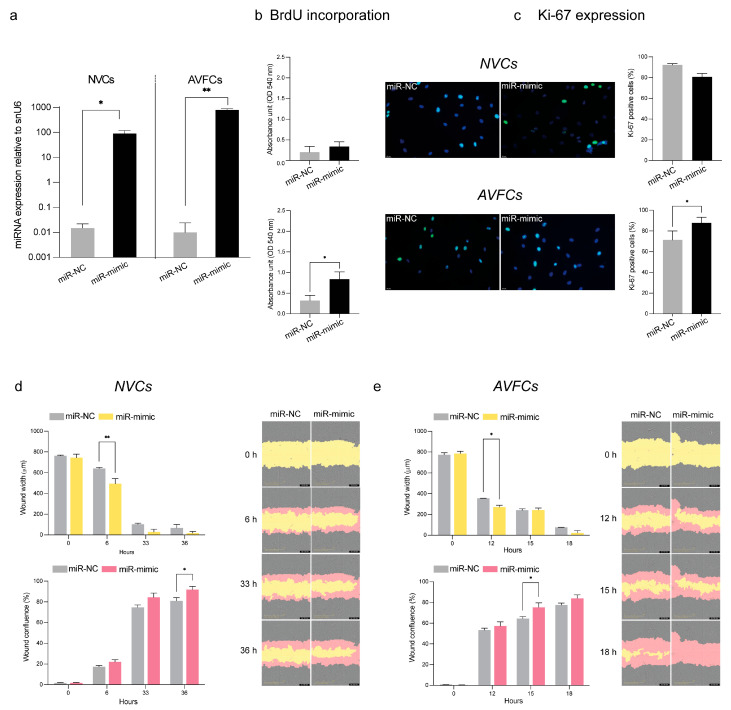
hsa-miR-449a-5p contributes to the main IH pathogenic mechanisms by stimulating the proliferative and migratory cell phenotype. (**a**) Validation of hsa-miR-449a-5p over-expression by transient transfection in NVCs and AVFCs after 48 h. Effects of hsa-miR-449a over-expression on NVCs and AVFCs’ proliferation, measured by (**b**) BrdU incorporation as a marker of newly synthesized DNA and (**c**) Ki-67 staining by immunofluorescence. For BrdU, absorbance values were analyzed at the 450 nm optical density (OD) with a reference wavelength of 595 nm by a Spark multimode microplate reader (Tecan). For Ki-67 expression, data are reported as the ratio of Ki-67 positive cell number to the total cell number. Green: Ki-67, blue: DAPI. Scale bar: 25 μm, 40× magnification. (**d**) Analysis of NVCs and (**e**) AVFCs’ migration performed through a scratch assay by using an Incucyte S3 instrument. The following migration parameters were evaluated: wound width, expressed in micrometers (μm), and wound confluence, expressed as a percentage (%). Representative images of wound healing taken with a 10× objective lens at the following time lapses: 0 h (initial wound process), 6 h, 33 h and 36 h for NVCs; 0 h, 12 h, 15 h and 18 h for AVFCs. Yellow: scratch wound width; pink: wound confluence (cells migrated into the scratch area). Data are reported as mean ± SD of at least three independent experiments. Statistical analysis was performed with an unpaired Student *t* test for (**a**–**c**), and a two-way ordinary ANOVA test followed by Sidak’s multiple-comparisons tests for (**d**,**e**); * *p* < 0.05; ** *p* < 0.01. Abbreviations: BrdU, bromo-deoxy-uridine; OD, optical density; miR-NC, negative control; miR-mimic, hsa-miR-449a-5p over-expression; NVCs, normal vein cells; AVFCs, arteriovenous fistula cells.

To deepen the knowledge of the role of hsa-miR-449a-5p in IH, we attempted to unveil its biological effects in vitro by focusing on patient-derived cell models. To this aim, we evaluated cell proliferation and migration following the over-expression of hsa-miR-449a-5p by transient transfection for 48 h (Figure 4a). The effects in terms of cell proliferation differed between NV and AVF cell models. Indeed, the hsa-miR-449a-5p mimic did not significantly influence NVC proliferation as it did in AVFCs, where a marked increase in the BrdU incorporation rate and Ki-67 expression was found (Figure 4b,c). Further, hsa-miR-449a-5p over-expression stimulated the migration process in both cell models, as supported by scratch assay results at 6 h and 36 h in NVCs (Figure 4d) and at 12 h and 15 h in AVFCs (Figure 4e).

Considering the noteworthy contribution of the inflammatory process to IH, we further analyzed inflammatory cytokines in the cell culture medium of AVFCs following hsa-miR-449a-5p over-expression. As shown in Supplementary Figure S1, a significant increase in IL-1β and TNF-α occurred (Supplementary Figure S1).

## 4. Discussion

Intimal thickening and stenosis seriously affect AVF patency. The identification of the main molecular players driving IH onset and progression will pave the way to promising diagnostic and therapeutic approaches for preventing AVF degeneration and reducing patient morbidity. miRNAs, as fine-tuners of gene expression, contribute to IH pathogenesis and can be easily detected in both human tissues and fluids, representing a promising source of disease markers.

In the present study, we attempted to identify a miRNA signature of AVF with IH lesions. The miRNA profiling and subsequent validation highlighted a panel of five miRNAs significantly dysregulated (hsa-let-7a and hsa-miR-29c-3p down-regulated; hsa-miR-155-5p, hsa-miR-194 and hsa-miR-449a-5p up-regulated) between normal veins (NVs) and AVFs. In the validation step, the AVF group was divided into two subgroups: AVFs with IH and AVFs with aneurysm, which represents a serious late complication of AVF failure [20]. The main significant changes in miRNA expression levels emerged between NVs and AVFs IH, suggesting that different molecular mechanisms govern aneurysm pathogenesis. Several triggers, like the prolonged shear stress, nitric oxide decrease and the unbalanced activity of matrix metalloproteinases (MMPs), may contribute to aneurysmal complication in AVFs [21]. To minimize potential bias and confounding issues, AVFs with aneurysm were analyzed separately by AVFs with IH for miRNA validation. Our results offer novel perspectives for investigating the miRNA contribution to aneurysm occurrence.

The in vitro study was performed to gain insights into the biological roles of the IH-miRNAs and elucidate possible pathogenic mechanisms. The same miRNA pattern except for hsa-miR-29c-3p was reflected in vitro. In this regard, to recapitulate the cell populations contributing to IH, we employed a panel of different cell culture models including patient ex vivo cell cultures (NVCs for cells isolated from the NV patient group and AVFCs for cells isolated from AVF patients), human aortic SMCs (HAOSMCs) and human endothelial cells (HUVECs). Among the miRNAs that significantly emerged from the NV vs. AVF comparison, hsa-miR-155-5p increase in AVF IH is in agreement with the literature, according to which miR-155-5p contributes to cardiovascular disease, atherosclerotic plaque instability [22] and IH in an AVF mouse model, by stimulating SMC proliferation and extracellular matrix (ECM) production [12].

An intriguing finding arises from hsa-miR-29c-3p, which showed a discrepancy between tissue and cell cultures, being down-regulated in AVF tissues and up-regulated in AVF cell models and in inflammatory-primed HAOSMCs and HUVECs. These data shed the light on the gap between cell cultures and the tissue of origin, based on the existence of different transcriptional networks and the loss of tissue heterogeneity in the in vitro model [23].

According to the literature, plasma levels of miR-29c associate with carotid thickness [24] and have anti-fibrotic effects by targeting genes codifying for collagens, integrins and matrix metalloproteinases [25]. However, tissue and subcellular localization may distinguish between miR-29c functions. On the other hand, miR-29c down-regulated human atherosclerotic plaques in diabetic patients, and its up-regulation in a diabetic rat prevented neointima formation [26]. Furthermore, we found a significant gender effect on miR-29c-3p expression, which resulted in its significantly lower expression in female subjects, who were more highly represented in the AVF group. Gender differences in miRNA expression have been observed in several pathological contexts [27]. This points out a limitation of the present study, which needs to be addressed by increasing the sample size and resolving the gender mismatch between NV and AVF groups.

According to our results, a significant up-regulation of hsa-miR-194b was found in the AVF IH group, but few studies have investigated its role in cardiovascular diseases so far. The pathway union analysis revealed the involvement of this miRNA in the Hippo signaling pathway, which is largely involved in atherosclerosis and restenosis following vascular interventions [28]. Further investigations are needed to deepen the understanding of the unexplored role of hsa-miR-194b in IH and AVF failure.

A focus was placed on hsa- miR-449a-5p, which caught our interest for its controversial role. Indeed, a broad range of evidence supports an anti-proliferative role of miR-449a-5p in cancer [29,30]. Otherwise, miR-449a-5p was shown to promote inflammation, endothelial cell dysfunction, proliferation, End-MT and vascular SMC phenotype transformation [31,32,33]. According to these findings, miR-449a-5p contributes to vascular disease, playing a leading role in atherosclerosis and plaque instability. Further, up-regulated expression of miR-449a-5p has been reported in plasma of patients with acute myocardial infarction [34]. These opposite findings indicate that miR-449a-5p is tightly context-specific, and our data are in accordance with the disease-promoting effects in the vascular system [30]. To deepen the knowledge of miR-449a-5p biology, we mimicked the over-expression of hsa-miR-449a-5p in vitro on patient cells from the NV and AVF groups, observing in both cell models a prominent increase in cell migration. Conversely, a deeper influence on cell proliferation was found in AVFCs, where hsa-miR-449a-5p further stimulated proliferation leading to a more aggressive cell phenotype. The different tissue origin might affect cell responsiveness to miRNA over-expression, explaining the different effect on cell proliferation between NV and AVF cells. Further, we explored the hsa-miR-449a-5p role in the inflammatory process, observing a significant increase of the inflammatory cytokines IL-1β and TNF-α in AVFCs following hsa-miR-449a-5p expression. These data bring novel insights into the hsa-miR-449a-5p function, supporting its role in IH pathogenesis and corroborating data obtained in AVF tissues.

An intriguing suggestion arises from the differential response to pioglitazone, which regulates cell proliferation and migration by inducing the nuclear hormone receptor PPAR-γ [16]. Pioglitazone belongs to the insulin-sensitizing agents thiazolidinediones (TDZs) and is an approved drug for type 2 diabetes. However, a large spectrum of studies support pioglitazone off-label indications and its beneficial effects on the cardiovascular system, including reducing effects on fibrosis and vascular remodeling [35]. According to our experiments, pioglitazone down-regulated hsa-miR-155-5p and hsa-miR-29c-3p in all cell models, whereas hsa-miR-449a-5p was further up-regulated following treatment in AVFCs and HAOSMCs. These data depict the pharmacological modulation of the selected miRNAs as a possible strategy for regulating the main mechanisms involved in IH pathogenesis and, as consequence, AVF failure. Implementing miRNA analysis at multiple time points would clarify their regulatory dynamics and the specific contribution to IH pathogenesis in vitro. In addition, further studies are still needed to fully explain the regulatory mechanisms behind hsa-miR-449a-5p up-regulation under pioglitazone administration.

The small study population and the gender mismatch are critical study limitations that need to be overcome by increasing the size of the sample group. Another limitation of the present study is the clinical setting of CKD pre-dialytic patients, characterized by uremia, inflammation, hypertension and dyslipidemia, well-known factors that may affect the genic and molecular pattern of vascular cells, including the histologically normal veins used to perform AVF.

## 5. Conclusions

The present study identified a miRNA panel distinctive of failed AVF carrying IH lesions. The most significant deregulated miRNAs are involved in focal adhesion, p53, FoxO and Hippo signaling pathways. Among the deregulated miRNAs, we focused on hsa-miR-449a-5p, which demonstrated an enhancing effect on proliferation and migration in vitro but was not sensitive to pioglitazone modulation. The involvement of hsa-miR-449a-5p in IH and the possible connection with PPAR-γ expression deserve deepen investigations.

Unveiling the most important miRNA networks associated with IH pathogenesis will support future investigations for planning innovative approaches addressed at the prevention of AVF failure, risk stratification with novel staging markers and IH lesion modulation. The evaluation of this miRNA panel in body fluids, such as patient serum, will be an important step for clarifying the miRNA involvement in the IH pathogenesis and AVF failure, and as such it has a possible application in the clinical setting for the detection and monitoring of failure and degeneration.

## Figures and Tables

**Table 1:** Clinical characteristics of the study population.

Demographic and Clinical Data	Patient Group	
	NV (*n* = 8)	AVF (*n* = 8)
Mean age	69 ± 16	60 ± 12
Males	7	4
Females	1	7
Diabetes	0	1
Hypertension	7	10
Vein fibrosis	1	1
Vein stenosis	0	1

NV, normal vein patient group; AVF, arteriovenous fistula revision patient group.

**Table 2:** Fold change values of miRNAs differently regulated between NV and AVF groups.

		Array Card		Single Assay qPCR	
miRNA Assay Name	miRBase Accession Number	Fold Change AVF vs. NV	*p* Value	Fold Change AVF vs. NV	*p* Value
hsa-miR-155-5p	MIMAT0000646	3.77	0.002	2.930	0.002
hsa-miR-449a-5p	MIMAT0001541	5.319	0.025	2.945	0.03
hsa-miR-29c-3p	MIMAT0000681	0.055	0.036	0.870	0.01
hsa-miR-194-5p	MIMAT0000460	5.388	0.044	3.38	0.003

NV, normal vein; AVF, intimal hyperplasia and aneurysm veins subjected to arteriovenous fistula revision.

## Data Availability

All relevant data are available within the manuscript.

## Supplementary Materials

The following supporting information can be downloaded at https://www.mdpi.com/article/10.3390/biom15081064/s1, Table S1. List of miRNA ID and sequences used for RT-qPCR. Table S2. Fold changes of deregulated miRNAs obtained from microfluidic cards profiling with a relative *p*-value <0.2. Table S3. Analysis of KEGG pathways generated by DIANA mirRPath v4.0 merged by pathways union for the 4 miRNAs selected. Abbreviations: KEGG, Kyoto Encyclopedia of Genes and Genomes. Table S4. Analysis of Gene Ontology enrichment analysis of Biological Processes (GO:BP) generated by DIANA mirRPath v4.0 merged by pathways union for the 4 selected miRNAs. Table S5. miRNET enrichment analysis in the KEGG dataset for the for the 4 selected miRNAs. Table S6. miRNET enrichment analysis in the GO dataset (GO:BP) for the 4 selected miRNAs. Figure S1. miR-449a-5p stimulates AVFCs T1 secretion of IL-1b and TNF-a. Extracellular levels of IL-1b, IL-6, TNF-a and IL-8 were measured in cell culture medium after 48h miR-449a-5p over-expression, by immunoassay performed with fully automated immunoassay platform ELLA (ProteinSimple/Bio-techne). Data are reported as mean ± standard deviation of three technical replicates obtained from two independent experiments and expressed as pg/ml. miR-NC, miRNA negative control; miR-mimic, miR-449a-5p overexpression.

## Abbreviations

The following abbreviations are used in this manuscript:

AVF	arteriovenous fistula
AVFCs	arteriovenous fistula cells
CKD	chronic kidney disease
FDR	false discovery rate
HAOSMCs	human aortic smooth muscle cells
KEGG	Kyoto Encyclopedia of Genes and Genomes
IH	intimal hyperplasia
miRNA	microRNA
NV	normal veins
NVCs	normal vein cells
