# Supplementary material for: A Distinct miRNA Profile in Intimal Hyperplasia of Failed Arteriovenous Fistulas Reveals Key Pathogenic Pathways
Source: Biomolecules. 2025 Jul 23;15(8):1064. doi: 10.3390/biom15081064 (PMC12383664; doi:10.3390/biom15081064)
Supplement: Supplementary file 1 [file biomolecules-15-01064-s001.zip › biomolecules-3682862-supplementary.pdf]

Table S1. List of miRNA ID and sequences used for RT-qPCR.

miRNA	Assay ID	Sequence
miR-155-5p	002623	UAAUGCUAAUCGUGAUAGGGGU
miR-449a-5p	001030	UGGCAGUGUAUUGUUAGCUGGU
miR-29c-3p	000587	UAGCACCAUUUGAAAUCGGUUA
miR-194-5p	000493	UGUAACAGCAACUCCAUGUGGA

Table S2. Fold changes of deregulated miRNAs obtained from microfluidic cards profiling with a relative p-value <0.2.

miRNA ID	Assay ID	Fold change (AVF vs NV)	p value
hsa-miR-155-5p	002623	3,777	*0,002
hsa-miR-449a	001030	5,319	*0,025
hsa-miR-29c-3p	000587	0,055	*0,036
hsa-let-7a	000377	0,195	*0,042
hsa-miR-194-5p	000493	5,388	*0,044
hsa-miR-216a	002220	2,949	0,052
hsa-miR-486-3p	002093	5,287	0,070
hsa-miR-146b	001097	6,511	0,074
hsa-miR-32	002109	7,141	0,085
hsa-miR-296-3p	002101	0,007	0,114
hsa-miR-215	000518	2,107	0,117
hsa-miR-383	000573	0,011	0,131
hsa-miR-542-5p	002240	0,221	0,136
hsa-miR-411	001610	3,203	0,155
hsa-miR-433	001028	5,259	0,158
hsa-miR-362	001273	2,726	0,187
hsa-miR-486	001278	8,291	0,187
hsa-miR-519a	002415	3,539	0,189
hsa-miR-627	001560	0,424	0,194

Table S3. Analysis of KEGG pathways generated by DIANA mirRPath v4.0 merged by pathways union for the 4 miRNAs selected.

KEGG patwhay	miRNAs involved	*p*-value
Focal adhesion	hsa-miR-194-5p	4,11057E-12
	hsa-miR-29c-3p	
Colorectal cancer	hsa-miR-155-5p	1,18281E-11
	hsa-miR-29c-3p	
Pathways in cancer	hsa-miR-155-5p	6,87697E-11
	hsa-miR-29c-3p	
Small cell lung cancer	hsa-miR-155-5p	1,23931E-10
	hsa-miR-29c-3p	
Apoptosis	hsa-miR-155-5p	3,52652E-10
	hsa-miR-29c-3p	
Platinum drug resistance	hsa-miR-155-5p	8,69763E-10
	hsa-miR-29c-3p	
p53 signaling pathway	hsa-miR-29c-3p	1,17339E-09
	hsa-miR-449a	
FoxO signaling pathway	hsa-miR-155-5p	6,66782E-09
	hsa-miR-29c-3p	
Hepatitis C	hsa-miR-155-5p	9,77617E-09
	hsa-miR-29c-3p	
Pancreatic cancer	hsa-miR-155-5p	1,49674E-08
	hsa-miR-29c-3p	
Ubiquitin mediated proteolysis	hsa-miR-194-5p	2,51718E-08
	hsa-miR-155-5p	

PI3K-Akt signaling pathway	hsa-miR-29c-3p	3,41575E-08
AGE-RAGE signaling pathway in diabetic complications	hsa-miR-155-5p<br>hsa-miR-29c-3p	7,46071E-08
Cellular senescence	hsa-miR-29c-3p<br>hsa-miR-449a	9,56612E-08
Prolactin signaling pathway	hsa-miR-155-5p<br>hsa-miR-29c-3p	1,67518E-07
Viral carcinogenesis	hsa-miR-155-5p<br>hsa-miR-29c-3p	3,7917E-07
Amyotrophic lateral sclerosis	hsa-miR-194-5p	4,94977E-07
Prostate cancer	hsa-miR-155-5p<br>hsa-miR-29c-3p	4,92044E-07
Relaxin signaling pathway	hsa-miR-29c-3p	7,84489E-07
Protein digestion and absorption	hsa-miR-29c-3p	1,19476E-06
Neurotrophin signaling pathway	hsa-miR-29c-3p	1,41541E-06
Measles	hsa-miR-155-5p<br>hsa-miR-29c-3p	1,42654E-06
Apoptosis - multiple species	hsa-miR-29c-3p	1,60843E-06
Endocrine resistance	hsa-miR-155-5p<br>hsa-miR-29c-3p	1,89601E-06
Autophagy - animal	hsa-miR-29c-3p	2,31119E-06
Hepatitis B	hsa-miR-155-5p<br>hsa-miR-29c-3p	2,52646E-06
Oxidative phosphorylation	hsa-miR-194-5p	2,79634E-06
Adherens junction	hsa-miR-194-5p<br>hsa-miR-449a	2,87401E-06

Human papillomavirus infection	hsa-miR-29c-3p	
Kaposi sarcoma-associated herpesvirus	hsa-miR-155-5p	3,06161E-06
infection	hsa-miR-29c-3p	3,37658E-06
Chronic myeloid leukemia	hsa-miR-155-5p	
	hsa-miR-29c-3p	3,35846E-06
	hsa-miR-194-5p	
Endocytosis	hsa-miR-29c-3p	3,63657E-06
Shigellosis	hsa-miR-155-5p	6,11851E-06
Hippo signaling pathway	hsa-miR-29c-3p	9,92548E-06
Pathways of neurodegeneration - multiple	hsa-miR-194-5p	
diseases	hsa-miR-29c-3p	1,0091E-05
	hsa-miR-29c-3p	
Proteoglycans in cancer	hsa-miR-29c-3p	1,03113E-05
Endometrial cancer	hsa-miR-29c-3p	1,3492E-05
HIF-1 signaling pathway	hsa-miR-449a	1,62232E-05
Glioma	hsa-miR-29c-3p	1,81067E-05
Bacterial invasion of epithelial cells	hsa-miR-29c-3p	2,13776E-05
Hepatocellular carcinoma	hsa-miR-29c-3p	2,56018E-05
Salmonella infection	hsa-miR-155-5p	2,92901E-05
	hsa-miR-29c-3p	
Alzheimer disease	hsa-miR-194-5p	3,14571E-05
	hsa-miR-29c-3p	
Prion disease	hsa-miR-194-5p	3,21539E-05
Fluid shear stress and atherosclerosis	hsa-miR-29c-3p	3,35639E-05
Renal cell carcinoma	hsa-miR-29c-3p	6,95222E-05
Central carbon metabolism in cancer	hsa-miR-449a	0,000107366

Transcriptional misregulation in cancer	hsa-miR-155-5p	0,00011695
	hsa-miR-29c-3p	
Breast cancer	hsa-miR-29c-3p	0,000138858
Thermogenesis	hsa-miR-194-5p	0,00015121
Parkinson disease	hsa-miR-194-5p	0,000158578
ECM-receptor interaction	hsa-miR-29c-3p	0,000172423
mTOR signaling pathway	hsa-miR-29c-3p	0,000187196
Longevity regulating pathway	hsa-miR-29c-3p	0,00019433
Signaling pathways regulating pluripotency of stem cells	hsa-miR-29c-3p	0,000194487
Epstein-Barr virus infection	hsa-miR-155-5p	0,000209274
RNA degradation	hsa-miR-155-5p	0,000224069
MicroRNAs in cancer	hsa-miR-29c-3p	0,000300743
AMPK signaling pathway	hsa-miR-29c-3p	0,000329585
EGFR tyrosine kinase inhibitor resistance	hsa-miR-29c-3p	0,000401393
Non-alcoholic fatty liver disease	hsa-miR-29c-3p	0,000472359
Amoebiasis	hsa-miR-29c-3p	0,000475492
NF-kappa B signaling pathway	hsa-miR-155-5p	0,000595483
Biosynthesis of amino acids	hsa-miR-449a	0,00083428
Cell cycle	hsa-miR-29c-3p	0,000824393
Apelin signaling pathway	hsa-miR-29c-3p	0,00084398
Gastric cancer	hsa-miR-29c-3p	0,000848057
Insulin signaling pathway	hsa-miR-29c-3p	0,001005744
Cholinergic synapse	hsa-miR-29c-3p	0,001157152
Huntington disease	hsa-miR-194-5p	0,001203405
Insulin resistance	hsa-miR-29c-3p	0,001389108

Bladder cancer	hsa-miR-155-5p	0,001515558
	hsa-miR-29c-3p	
Thyroid hormone signaling pathway	hsa-miR-29c-3p	0,001666553
	hsa-miR-29c-3p	
Choline metabolism in cancer	hsa-miR-29c-3p	0,001894205
	hsa-miR-29c-3p	
ErbB signaling pathway	hsa-miR-29c-3p	0,002048529
Mitophagy - animal	hsa-miR-29c-3p	0,002062983
Melanoma	hsa-miR-29c-3p	0,002062983
Protein processing in endoplasmic reticulum	hsa-miR-155-5p	0,002800477
Lysine degradation	hsa-miR-29c-3p	0,002892445
JAK-STAT signaling pathway	hsa-miR-155-5p	0,002950835
Spinocerebellar ataxia	hsa-miR-29c-3p	0,003143463
TNF signaling pathway	hsa-miR-155-5p	0,003414153
Non-small cell lung cancer	hsa-miR-29c-3p	0,003562869
Yersinia infection	hsa-miR-29c-3p	0,003647791
Chagas disease	hsa-miR-29c-3p	0,004583116
Regulation of actin cytoskeleton	hsa-miR-29c-3p	0,0051111
Rap1 signaling pathway	hsa-miR-29c-3p	0,005829986
Dopaminergic synapse	hsa-miR-29c-3p	0,006273997
Aldosterone-regulated sodium reabsorption	hsa-miR-29c-3p	0,006359541
Sphingolipid signaling pathway	hsa-miR-29c-3p	0,007042932
Hypertrophic cardiomyopathy	hsa-miR-29c-3p	0,007056984
Fc gamma R-mediated phagocytosis		0,008331527
Pathogenic Escherichia coli infection		0,008991339
Progesterone-mediated oocyte maturation		0,010575338

Abbreviations: KEGG, Kyoto Encyclopedia of Genes and Genomes

Table S4. Analysis of Gene Ontology enrichment analysis of Biological Processes (GO:BP) generated by DIANA mirRPath v4.0 merged by pathways union for the 4 selected miRNAs.

GO Term Name	miRNAs involved	*p*-value
viral process	hsa-miR-194-5p<br>hsa-miR-155-5p<br>hsa-miR-29c-3p<br>hsa-miR-449a	3,88087E-30
negative regulation of transcription by RNA polymerase II	hsa-miR-155-5p<br>hsa-miR-29c-3p<br>hsa-miR-449a	9,088E-24
chromatin organization	hsa-miR-194-5p<br>hsa-miR-155-5p<br>hsa-miR-29c-3p<br>hsa-miR-449a	8,62248E-23
positive regulation of transcription by RNA polymerase II	hsa-miR-155-5p<br>hsa-miR-29c-3p	2,79742E-15
positive regulation of transcription, DNA-templated	hsa-miR-155-5p<br>hsa-miR-29c-3p	1,35487E-12
protein ubiquitination	hsa-miR-155-5p<br>hsa-miR-29c-3p	1,14047E-10

negative regulation of transcription, DNA-templated	hsa-miR-155-5p	2,44472E-10
extracellular matrix organization	hsa-miR-29c-3p	7,46324E-10
positive regulation of nuclear-transcribed mRNA poly(A) tail shortening	hsa-miR-155-5p hsa-miR-29c-3p	1,13124E-09
positive regulation of nuclear-transcribed mRNA catabolic process, deadenylation-dependent decay	hsa-miR-155-5p hsa-miR-449a	2,20963E-09
regulation of transcription, DNA-templated	hsa-miR-155-5p	5,41034E-08
peptidyl-serine phosphorylation	hsa-miR-155-5p	3,16327E-07
cellular response to DNA damage stimulus	hsa-miR-155-5p	5,16888E-07
cellular response to amino acid stimulus	hsa-miR-29c-3p	5,65614E-07
electron transport coupled proton transport	hsa-miR-194-5p	7,18701E-07
cell cycle	hsa-miR-155-5p	1,25097E-06
regulation of translation	hsa-miR-29c-3p	2,45085E-06

ATP synthesis coupled electron transport	hsa-miR-194-5p	4,87589E-06
intrinsic apoptotic signaling pathway in response to DNA damage	hsa-miR-29c-3p	5,9565E-06
isotype switching	hsa-miR-155-5p	5,89242E-06
response to drug	hsa-miR-29c-3p	6,69432E-06
hippo signaling	hsa-miR-155-5p	7,63753E-06
skin development	hsa-miR-29c-3p	8,42954E-06
regulation of transcription by RNA polymerase II	hsa-miR-155-5p	1,06486E-05
regulation of cell cycle	hsa-miR-449a	1,30715E-05
mitochondrial electron transport, NADH to ubiquinone	hsa-miR-194-5p	1,45964E-05
DNA damage response, signal transduction by p53 class mediator resulting in cell cycle arrest	hsa-miR-29c-3p	1,69041E-05

erythrocyte differentiation		1,66535E-05
post-translational protein modification		1,75356E-05
rhythmic process	hsa-miR-29c-3p	1,79102E-05
protein polyubiquitination	hsa-miR-155-5p	1,9301E-05
ubiquitin-dependent protein catabolic process	hsa-miR-155-5p	1,94987E-05
positive regulation of apoptotic process	hsa-miR-29c-3p	2,09109E-05
membrane organization	hsa-miR-194-5p	2,10171E-05
histone methylation	hsa-miR-29c-3p	2,49717E-05
negative regulation of cyclin-dependent protein serine/threonine kinase activity	hsa-miR-155-5p	2,45476E-05
regulation of cholesterol biosynthetic process	hsa-miR-29c-3p	2,62267E-05
negative regulation of translation	hsa-miR-29c-3p	2,93268E-05
protein phosphorylation	hsa-miR-155-5p	3,34108E-05
regulation of RNA splicing	hsa-miR-155-5p	4,23821E-05
skeletal system development	hsa-miR-29c-3p	4,62029E-05
positive regulation of endoplasmic reticulum unfolded protein response	hsa-miR-29c-3p	5,36024E-05
response to endoplasmic reticulum stress	hsa-miR-155-5p	5,54334E-05
nuclear-transcribed mRNA poly(A) tail shortening	hsa-miR-29c-3p	5,97371E-05
regulation of mRNA stability	hsa-miR-155-5p	5,8432E-05
protein import into nucleus	hsa-miR-155-5p	7,13547E-05
tissue development	hsa-miR-29c-3p	7,07562E-05
intracellular signal transduction	hsa-miR-155-5p	7,91079E-05

homeostasis of number of cells within a tissue	hsa-miR-155-5p	8,32075E-05
cell division	hsa-miR-29c-3p	8,45045E-05
gene silencing by RNA	hsa-miR-29c-3p	9,03932E-05
response to hydrogen peroxide	hsa-miR-29c-3p	0,000101419
post-embryonic development	hsa-miR-29c-3p	0,000104972
nuclear-transcribed mRNA catabolic process, deadenylation-dependent decay	hsa-miR-155-5p	0,000111171
establishment of endothelial intestinal barrier	hsa-miR-155-5p	0,000111171

Table S5. miRNET enrichment analysis in the KEGG dataset for the for the 4 selected miRNAs.

**Pathway**	**Total**	**Expected**	**Hits**	***p*-value**	**FDR**
Aminoacyl-tRNA biosynthesis	7	4.39	9	0	0
Pathways in cancer	310	194	248	7.98e-12	3.99e-10
Axon guidance	118	74	103	1.57e-09	5.23e-08
HTLV-I infection	199	125	160	2.69e-08	6.72e-07
Focal adhesion	200	125	160	5.21e-08	1.04e-06
RNA transport	126	79	106	7.6e-08	1.27e-06
Cell cycle	124	77.7	104	1.4e-07	2E-06
Protein processing in endoplasmic reticulum	129	80.9	107	2.99e-07	3.74e-06
p53 signaling pathway	68	42.6	61	4.36e-07	4.84e-06

Regulation of actin cytoskeleton	182	114	144	8.77e-07	8.77e-06
Adherens junction	70	43.9	62	1.03e-06	9.36e-06
Small cell lung cancer	80	50.2	69	2.37e-06	1.98e-05
Prostate cancer	87	54.5	74	3.2e-06	2.46e-05
Influenza A	107	67.1	88	7.09e-06	5.06e-05
Citrate cycle (TCA cycle)	30	18.8	29	1.49e-05	9.31e-05
Oocyte meiosis	108	67.7	88	1.49e-05	9.31e-05
Chronic myeloid leukemia	73	45.8	62	2.29e-05	0.000131
Pancreatic cancer	69	43.3	59	2.35e-05	0.000131
N-Glycan biosynthesis	43	27	39	3.29e-05	0.000173
Insulin signaling pathway	137	85.9	107	6.49e-05	0.000324
Progesterone-mediated oocyte maturation	80	50.2	66	8.46e-05	0.00039
Colorectal cancer	49	30.7	43	8.58e-05	0.00039
Endocytosis	101	63.3	81	9.75e-05	0.000424
Renal cell carcinoma	60	37.6	51	0.00012	0.000477
Bacterial invasion of epithelial cells	56	35.1	48	0.000123	0.000477
Phosphatidylinositol signaling system	75	47	62	0.000124	0.000477
Lysine degradation	47	29.5	41	0.000171	0.000633
Glioma	65	40.8	54	0.000265	0.000946
Toxoplasmosis	93	58.3	74		0.00108

Neurotrophin signaling pathway	123	77.1	95	0.000349	0.00116
MAPK signaling pathway	265	166	192	0.000363	0.00117
RNA degradation	60	37.6	50	0.000388	0.00118
Jak-STAT signaling pathway	99	62.1	78	0.000388	0.00118
Non-small cell lung cancer	52	32.6	44	0.000443	0.0013
Valine, leucine and isoleucine degradation	44	27.6	38	0.000472	
Circadian rhythm - mammal	22	13.8	21	0.000476	0.00132
Wnt signaling pathway	144	90.3	109	0.000525	0.00142
ErbB signaling pathway	87	54.5	69	0.000592	0.00156
Inositol phosphate metabolism	58	36.4	48	7E-04	0.00179
Alzheimer's disease	49	30.7	41	0.00112	0.0028
mTOR signaling pathway	45	28.2	38	0.0012	0.00293
Endometrial cancer	44	27.6	37	0.00164	0.0039
Huntington's disease	28	17.6	25	0.00172	4
	72	45.1	57	0.00192	0.00436
Pyrimidine metabolism	101	63.3	77	0.00242	0.00538
Acute myeloid leukemia	57	35.7	46	0.00255	0.00554
RIG-I-like receptor signaling pathway	49	30.7	40	0.00327	0.00696
Glycerolipid metabolism	52	32.6	42	0.00381	0.00794
mRNA surveillance pathway	82	51.4	63	0.00429	0.00876

Prion diseases	21	13.2	19	0.00477	0.00954
Bladder cancer	29	18.2	25	0.00504	0.00974
Epstein-Barr virus infection	91	57	69	0.00508	0.00974
Alcoholism	166	104	120	0.00516	0.00974
Notch signaling pathway	47	29.5	38	0.00569	0.0105
Herpes simplex infection	103	64.6	77	0.00598	0.0109
Long-term potentiation	70	43.9	54	0.0069	0.0123
Dopaminergic synapse	124	77.7	91	0.00718	0.0126
Gap junction	89	55.8	67	0.00766	0.0132
Pathogenic Escherichia coli infection	35	21.9	29	0.00816	0.0138
SNARE interactions in vesicular transport	27	16.9	23	0.00974	0.0162
Ribosome biogenesis in eukaryotes	55	34.5	43	0.0103	0.0166
Fc gamma R-mediated phagocytosis	97	60.8	72	0.0103	0.0166
ECM-receptor interaction	84	52.7	63	11	0.0175
Pyruvate metabolism	41	25.7	33	0.0112	0.0175
Glycosphingolipid biosynthesis - ganglio series	14	8.78	13	0.0134	0.0197
Apoptosis	83	52	62	0.0134	0.0197
beta-Alanine metabolism	26	16.3	22	0.0134	0.0197
Viral myocarditis	26	16.3	22	0.0134	0.0197

Chagas disease (American trypanosomiasis)	89	55.8	66	0.0143	0.0206
Propanoate metabolism	22	13.8	19	0.0144	0.0206
Legionellosis	40	25.1	32	0.0146	0.0206
Arginine and proline metabolism	56	35.1	43	0.0176	0.0244
Sphingolipid metabolism	46	28.8	36	0.0179	0.0245
Hepatitis C	100	62.7	73	0.0186	0.0248
B cell receptor signaling pathway	75	47	56	0.0186	0.0248
Fanconi anemia pathway	39	24.4	31	19	25
Alanine, aspartate and glutamate metabolism	32	20.1	26	0.0195	0.0253
Tight junction	118	74	85	0.0197	0.0253
Thyroid cancer	28	17.6	23	0.0222	0.0281
Regulation of autophagy	8	5.02	8	0.0238	0.0298
Amphetamine addiction	64	40.1	48	0.0251	31
Cholinergic synapse	95	59.6	69	0.0258	0.0315
Cytosolic DNA-sensing pathway	20	12.5	17	0.0279	0.0336
Purine metabolism	163	102	114	0.0298	0.0352
Selenocompound metabolism	12	7.52	11	0.0299	0.0352
Shigellosis	47	29.5	36	0.0307	0.0357
Epithelial cell signaling in Helicobacter pylori infection	37	23.2	29	0.0317	0.0364

TGF-beta signaling pathway	84	52.7	61	0.0351	0.0399
Cocaine addiction	43	27	33	0.0364	0.0409
Proximal tubule bicarbonate reclamation	7	4.39	7	38	0.0422
Vibrio cholerae infection	19	11.9	16	0.0386	0.0424
Synaptic vesicle cycle	18	11.3	15	0.0529	0.0575
Aldosterone-regulated sodium reabsorption	34	21.3	26	65	0.0699
Pentose phosphate pathway	27	16.9	21	0.0735	0.0782
Fructose and mannose metabolism	36	22.6	27	0.0844	0.0888
Amyotrophic lateral sclerosis (ALS)	39	24.4	29	0.0867	0.0903
Fatty acid elongation	23	14.4	18	0.0883	91
Type II diabetes mellitus	48	30.1	35	0.0911	93
T cell receptor signaling pathway	98	61.4	68	0.0992	0.1
Transcriptional misregulation in cancer	19	11.9	15	107	107

Table S6. miRNET enrichment analysis in the GO dataset (GO:BP) for the 4 selected miRNAs.

**Pathway**	**Total**	**Expected**	**Hits**	***p*-value**	**FDR**
interaction with host	426	271	346	8.91e-16	8.91e-14
viral reproductive process	597	380	452	9.89e-11	4.94e-09

negative regulation of cellular protein metabolic process	463	295	354	1.53e-09	5.1e-08
epidermal growth factor receptor signaling pathway	167	106	138	5.44e-08	1.36e-06
intracellular protein transport	793	505	572	1.5e-07	3E-06
G2/M transition of mitotic cell cycle	150	95.6	124	2.46e-07	3.61e-06
interphase of mitotic cell cycle	435	277	326	2.53e-07	3.61e-06
negative regulation of protein metabolic process	540	344	398	3.09e-07	3.79e-06
interphase	443	282	331	3.41e-07	3.79e-06
protein polyubiquitination	177	113	143	4.91e-07	4.37e-06
post-Golgi vesicle-mediated transport	79	50.3	70	5.36e-07	4.37e-06
regulation of cell morphogenesis	325	207	248	5.8e-07	4.37e-06
cell division	507	323	374	6.06e-07	4.37e-06
vacuole organization	65	41.4	59	6.12e-07	4.37e-06
protein transport	1400	893	973	1.17e-06	7.8e-06
maintenance of protein location in cell	101	64.4	86	1.43e-06	8.94e-06
maintenance of location in cell	112	71.4	94	1.83e-06	1.08e-05
establishment of protein localization	1460	927	1010	2.59e-06	1.44e-05
positive regulation of cell cycle	113	72	94	4.08e-06	2.15e-05
regulation of axonogenesis	104	66.3	87	5.86e-06	2.93e-05
maintenance of protein location	116	73.9	95	1.38e-05	6.57e-05
axon guidance	394	251	289	2.4e-05	0.000109
regulation of cell growth	302	192	225	3.51e-05	0.000153
peptidyl-amino acid modification	739	471	521	3.87e-05	0.000161
protein export from nucleus	48	30.6	43	4.97e-05	0.000199

S phase	153	97.5	120	5.7e-05	0.000219
post-translational protein modification	196	125	150	7.39e-05	0.000262
Golgi vesicle transport	206	131	157	7.43e-05	0.000262
axonogenesis	578	368	411	7.7e-05	0.000262
G1/S transition of mitotic cell cycle	209	133	159	7.9e-05	0.000262
nuclear transport	392	250	285	8.13e-05	0.000262
S phase of mitotic cell cycle	144	91.8	113	8.94e-05	0.000275
nucleocytoplasmic transport	388	247	282	9.19e-05	0.000275
histone modification	312	199	230	9.34e-05	0.000275
endoplasmic reticulum unfolded protein response	93	59.3	76	0.000115	0.000329
ER-nucleus signaling pathway	111	70.7	89	0.00012	0.000333
protein localization	1850	1180	1250	0.000139	0.000376
negative regulation of transferase activity	189	120	144	0.000149	0.000392
protein targeting to membrane	158	101	122	0.000173	0.000444
covalent chromatin modification	320	204	234	0.000191	0.000474
cytoskeleton-dependent intracellular transport	76	48.4	63	0.000197	0.000474
chromatin modification	512	326	364	0.000199	0.000474
regulation of mitotic cell cycle	351	224	255	0.000205	0.000477
cellular membrane organization	471	300	336	0.00023	0.000523
lysosomal transport	39	24.8	35	0.000242	0.000538
cellular response to extracellular stimulus	149	94.9	115	0.000272	0.000575
establishment of organelle localization	125	79.6	98	0.000274	0.000575
organelle localization	189	120	143	0.000276	0.000575
viral reproduction	803	512	557	0.000308	0.000629

phosphatidylinositol-mediated signaling	148	94.3	114	0.000333	0.000666
nuclear export	139	88.6	107	0.000524	0.00102
mitotic cell cycle checkpoint	149	94.9	114	0.000532	0.00102
G1 phase	49	31.2	42	0.000575	0.00108
nucleobase-containing compound transport	175	112	132	0.000585	0.00108
protein targeting	545	347	383	0.000593	0.00108
nuclear import	232	148	171	0.000714	0.00128
regulation of cyclin-dependent protein kinase activity	89	56.7	71	0.00076	0.00132
protein complex assembly	861	549	592	0.000775	0.00132
focal adhesion assembly	44	28	38	0.000776	0.00132
regulation of translation	228	145	168	0.000811	0.00133
protein import into nucleus	228	145	168	0.000811	0.00133
mitosis	420	268	298	0.000895	0.00144
negative regulation of translation	70	44.6	57	0.000973	0.00154
Ras protein signal transduction	274	175	199	1	0.00156
response to ionizing radiation	112	71.4	87	0.00102	0.00157
G1 phase of mitotic cell cycle	47	29.9	40	0.00106	0.00161
protein oligomerization	357	227	255	0.00111	0.00165
cellular protein catabolic process	518	330	363	0.00112	0.00165
response to drug	344	219	246	0.00121	0.00175
viral infectious cycle	241	154	176	0.00125	0.00179
lysosome organization	34	21.7	30	0.00135	0.0019
negative regulation of cellular component organization	370	236	263	0.00148	2
negative regulation of phosphorylation	246	157	179	0.00152	2

regulation of neuron apoptotic process	150	95.6	113	0.00153	2
regulation of growth	548	349	382	0.00153	2
regulation of cellular component size	192	122	142	0.00154	2
maintenance of location	192	122	142	0.00154	
vesicle localization	82	52.2	65	0.00172	0.0022
regulation of nucleocytoplasmic transport	172	110	128	0.00174	0.0022
cellular response to nutrient levels	126	80.3	96	0.00181	0.00226
vacuolar transport	45	28.7	38	0.00192	0.00237
regulation of viral reproduction	122	77.7	93	0.00206	0.00251
macromolecular complex assembly	1120	715	759	0.00227	0.00273
regulation of protein kinase activity	698	445	480	0.00232	0.00276
positive regulation of I-kappaB kinase/NF-kappaB cascade	150	95.6	112	0.00271	0.00319
neuron apoptotic process	169	108	125	0.00285	0.00331
DNA damage checkpoint	143	91.1	107	0.00296	0.0034
establishment or maintenance of cell polarity	123	78.4	93	0.00318	0.00361
mitochondrial membrane organization	47	29.9	39	0.00323	0.00363
DNA integrity checkpoint	152	96.9	113	0.00334	0.00371
sister chromatid segregation	57	36.3	46	0.00421	0.00461
cellular component disassembly involved in execution phase of apoptosis	78	49.7	61	0.00424	0.00461
negative regulation of phosphate metabolic process	293	187	208	0.0048	0.00513
transcription initiation from RNA polymerase II promoter	219	140	158	0.00487	0.00513
viral genome replication	67	42.7	53	0.00487	0.00513

regulation of organelle organization	589	375	405	0.00496	0.00517
cellular macromolecule catabolic process	849	541	576	0.00524	0.0054
cytokinesis	120	76.5	90	0.00548	0.00559
growth	839	535	569	0.00575	0.00581
response to hypoxia	245	156	175	0.00619	0.00619

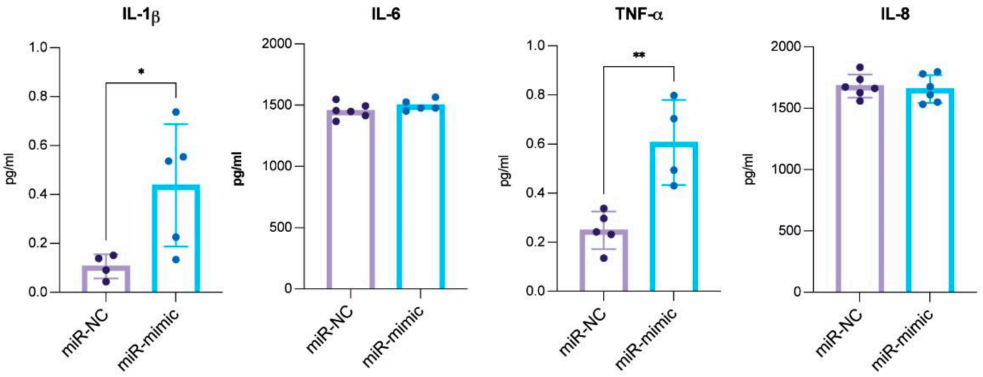

Figure S1. miR-449a-5p stimulates AVFCs T1 secretion of IL-1β and TNF-α. Extracellular levels of IL-1β, IL-6, TNF-α and IL-8 were measured in cell culture medium after 48h miR-449a-5p over-expression, by immunoassay performed with fully automated immunoassay platform ELLA (ProteinSimple/Bio-techne). Data are reported as mean ± standard deviation of three technical replicates obtained from two independent experiments and expressed as pg/ml. miR-NC, miRNA negative control; miR-mimic, miR-449a-5p over-expression.
